# The Effect of COVID-19 Vaccine Acceptance, Intention, and/or Hesitancy and Its Association with Our Health and/or Important Areas of Functioning

**DOI:** 10.3390/vaccines11020368

**Published:** 2023-02-06

**Authors:** Daniel Kwasi Ahorsu, Chung-Ying Lin

**Affiliations:** 1 Department of Rehabilitation Sciences, Faculty of Health & Social Sciences, The Hong Kong Polytechnic University, 11 Yuk Choi Rd Hung Hom, Hong Kong, China; 2 Mental Health Research Centre, The Hong Kong Polytechnic University, 11 Yuk Choi Rd Hung Hom, Hong Kong, China; 3 Institute of Allied Health Sciences, College of Medicine, National Cheng Kung University, Tainan 701, Taiwan

## Introduction

The emergence of coronavirus 2019 (COVID-19) has had a significant negative impact on the world, with its effect noted in various areas, such as commerce [1,2], education [3,4], health [5,6,7], and social life [8,9]. This life-threatening virus, which was first reported in Wuhan (China), was deemed to have a high fatality and infection rate [10,11]. This led the World Health Organisation (WHO) to classify it as a pandemic within three months, on 11th March 2020 [10,11]. Since then, countries worldwide attempted to prevent and/or contain COVID-19 by setting up COVID-19 guidelines or policies such as quarantining, hand washing, and physical distancing [12]. Although effective vaccines have been developed, the negative consequences of COVID-19 remain important issues worldwide. As of 10 January 2023, there had been over 660.1 million COVID-19 cases and over 6.6 million deaths globally [13]. Out of these cases, 270.5 million cases were in Europe, 187 million in the Americas, 109 million cases in the Western Pacific, 60.7 million in Southeast Asia, 23.2 million in the Eastern Mediterranean, and 9.4 million in Africa [13].

The search for an anti-virus (or vaccine) commenced with much urgency, with the first vaccine being approved on 31 December 2020 [14]. As of 12 January 2022, nine vaccines have obtained WHO Emergency Use Listing, including the Pfizer/BioNTech Comirnaty vaccine (31 December 2020); SII/COVISHIELD and AstraZeneca/AZD1222 vaccines (16 February 2021); Janssen/Ad26.COV 2.S vaccine (by Johnson & Johnson, New Jersey, US, 12 March 2021); Moderna COVID-19 vaccine (mRNA 1273, 30 April 2021); Sinopharm COVID-19 vaccine (7 May 2021); Sinovac-CoronaVac vaccine (1 June 2021); Bharat Biotech BBV152 COVAXIN vaccine (3 November 2021); Covovax (NVX-CoV2373) vaccine (17 December 2021); and Nuvaxovid (NVX-CoV2373) vaccine (20 December 2021) [14]. As the only intervention for COVID-19 is the vaccine and behavioural preventive practices, countries worldwide have been conducting studies to better understand their citizens’ attitude toward the vaccination and its association with other COVID-19-related variables; social, and/or occupational functioning; health; and other vaccines already in the system. As of 22 December 2022, 13.07 billion doses of vaccines have been administered globally [13]. Out of these 13.07 billion doses, the number of persons vaccinated with the last dose of the primary series was 438.7 million in Europe, 636.8 million in the Americas, 1.7 billion in the Western Pacific, 693.2 million in Southeast Asia, 346.9 million in the Eastern Mediterranean, and 273.6 million in Africa [15].

In the quest to understand and improve the COVID-19 vaccination drive, some researchers assume that some COVID-19-related variables—such as COVID-19 stress, fear of COVID-19, perceived stigma from COVID-19, self-stigma from COVID-19, and believing COVID-19 information—may be helpful in this regard [16,17,18]. For instance, a study by Adjaottor et al. [19] reported that believing COVID-19 information, danger and contamination fears, and traumatic stress (subscales of COVID-19 stress) were significant predictors of COVID-19 vaccination acceptance but not fear of COVID-19, perceived stigma from COVID-19, self-stigma from COVID-19, and COVID-19 infection prevention behaviours. Other researchers have reported interesting findings after examining the association between COVID-19-related variables and COVID-19 vaccination, taking into consideration sex, migration status, and others [20,21].

The mode of transmission of COVID-19 together with its high fatality rate led to the institution of preventive measures by governments, which impacted negatively on our functioning. First, there were lockdowns and quarantining, which restricted people’s movement and, consequently, their social, school, and/or occupational functioning [22,23,24]. Later, as lockdown measures were lifted, physical (social) distancing measures and the compulsory wearing of masks were introduced, which further limited social interactions and access to work/school spaces. This shortfall/challenges accelerated the use of the Internet or online mediums for teaching–learning, working, and social media interactions. Although these preventive measures were intended to contain and prevent the further transmission of COVID-19 infection, they also destabilised the normal functioning of society [22,23,24]. COVID-19 vaccination was, therefore, seen as one of the possible methods of returning to a state of normalcy. Earlier researchers suggested at least a 70% vaccination record to achieve herd immunity [25]. However, until now, there have been challenges with the vaccination drive. These include vaccination hesitancy and/or the unavailability/inadequacy of COVID-19 vaccines. Closely linked with vaccination challenges is the mutation of the COVID-19 virus limiting, the efficacy of the vaccines. Therefore, future studies may be needed to enhance our understanding of the vaccination drive on our functioning.

The debilitating effect of COVID-19 on physical and mental health has been reported worldwide. There have been various reports of physical health challenges such as fever, cough, breathing difficulty, sore throat, and gastrointestinal symptoms associated with post-COVID-19 infection [26,27]. Moreover, depression, anxiety, insomnia, and substance use disorders have been associated with post-COVID-19 infection [28,29]. There have also been reports of the higher likelihood of older people and people with chronic health conditions (e.g., hypertension and chronic obstructive pulmonary disease) to succumb to COVID-19 infection and mortality [30,31,32]. A comparative study on physical and mental health outcomes between vaccinated and unvaccinated COVID-19 participants with respect to post-COVID-19 infection may squash vaccination hesitancy and push forward COVID-19 vaccination. There is also a need to study some of the side effects of vaccinations and how to appropriately deal with them.

Prior to the emergence of COVID-19, there were several different types of vaccination drives. Taking this into account, some researchers believed that health officers could utilize these previous vaccination programmes to enhance the COVID-19 vaccination drive. This is also because the influenza vaccination was reported to be negatively associated with COVID-19 infection and mortality [33,34], and both vaccinations improved the physical quality of life [35]. However, a previous study examining the difference between COVID-19 and flu vaccination programmes revealed that flu vaccination rates have been affected after COVID-19 vaccination [36]. A study among the Hungarian population revealed that more participants were willing to receive a COVID-19 vaccine compared to seasonal influenza, even after grouping participants based on demographic data or perceived financial status [37]. Other studies provided different reasons and factors influencing COVID-19 vaccination [19,38,39,40]. Taking these studies into consideration, there will be a need for more studies examining the intention of receiving different vaccines and attitudes towards those vaccines and the vaccination drive.

In general, the literature above clearly indicates that several factors influence COVID-19 vaccination acceptance. Moreover, COVID-19 vaccination opens the way for us to function socially and occupationally, especially as the COVID-19 vaccine process is ongoing. Furthermore, as some countries are trying to declare COVID-19 as endemic [41,42,43], some scholars are discussing whether we should treat the COVID-19 vaccine as a flu vaccine. In addition, informational fatigue and misinformation continue to be a challenge. Therefore, COVID-19 vaccine acceptance should be contentiously investigated worldwide. Accordingly, the present Special Issue welcomes any type of investigation on the COVID-19 vaccine through the lens of psychosocial aspects, in order to help the scientific community better understand the issue of the COVID-19 vaccine and vaccination drive.
